# The Medical Association of the State of Alabama

**Published:** 1888-05

**Authors:** 


### Society Correspondence.

### THE MEDICAL ASSOCIATION OF THE STATE OF ALABAMA.

*Editors Atlanta Medical and Surgical Journal:*

The four days' session of the Medical Association of the State of Alabama, which is being held here and adjourned this evening, has been a successful one and adds additional lustre to the high position it already holds among its sister societies of the South.

That you may judge more fully of the merits of the matter presented a programme is given below:

### PROGRAMME.

FIRST DAY—TUESDAY, APRIL 10TH.

1. Registration at the court-room from 10 a. m. to 12 m.

2. The Association called to order by the President at 12 m. precisely.

3. Prayer by the Rev. Horace Stringfellow, D. D., of St. John's Church.

4. Address of welcome on behalf of the Medical and Surgical Society of Montgomery county by Dr. W. G. Bibb.

5. The annual message of the President, Dr. Edward H. Sholl, of Birmingham.

6. The annual report of the senior Vice-President, Dr. Thad. L. Robertson, of Birmingham.

7. The annual report of the junior Vice-President, Dr. John Paul Jones, of Camden.

8. Revision of the minutes of the proceedings of the session of 1887.

9. The report of the Secretary.

10. The report of the Treasurer.

11. The business reports of County Societies.

12. Reports of Special Committees.

13. Miscellaneous business.

Adjournment at 2:45 o'clock.

EVENING SESSION—8 O'CLOCK.

14. The annual oration by Dr. B. J Baldwin, of Montgomery, at the Montgomery Theatre. After the oration the audience will be entertained with recitations, readings and vocal and instrumental music by distinguished talent.

### SECOND DAY—WEDNESDAY, 11TH.

1. Registration.
2. The Association called to order at 9:45 a. m. precisely.
3. Unfinished and miscellaneous business.
4. The regular reports in the alphabetical order of their arrangement, viz.:
5. The Value of Exploratory Incisions—William Locke Chew, M. D., Birmingham.
6. The Radical Cure of Hernia—Luther Leonidas Hill, M. D., Montgomery.
7. The Practical Investigation as to the Therapeutic Value of Salicylate of Hydrargyrum—Henry Tutwiler Inge, M. D., Mobile.
8. Studies in Eczema—Arnold Jolly, M. D., Linden.
9. Electricity in Uterine Diseases—Mortimer Harvey Jordan, M. D., Birmingham.
10. Physiological and Pathological Heredity—James Thomas Searcy, M. D., Tuscaloosa.
11. Diarrhœal Diseases of Children in Western and Middle Alabama—Wm. Henry Sledge, M. D. Livingston.
12. The Medical Topography of Walker County and its Prevailing Diseases—Alexander McAdams Stovall, M. D., Jasper.
13. Color Blindness and Defective Vision in Relation to the Traveling Public and the Railroads—Robert Dickens Webb, M. D., Birmingham.
14. Studies in Rectal Surgery—Benjamin Leon Wyman, M. D., Birmingham.

Adjournment at 2:45 p. m.

### EVENING SESSION—7 O'CLOCK.

15. Discussion of the best methods of administering the health laws and the law to regulate the practice of medicine.

Adjournment at 9 o'clock.

Reception at the City Hall by the Medical and Surgical Society of Montgomery County from 9 to 12 p. m.

### THIRD DAY—THURSDAY, 12TH.

1. Registration.
2. The Association called to order at 9:45 a. m.
3. Unfinished and miscellaneous business.
4. The Omnibus Discussion, Milton Columbus Baldridge, M. D., of Huntsville, leader. Topics: 1st. Typho-Malarial Fever. 2nd. Hereditary Diseases. 3rd. Influenza. 4th. Pain as a Symptom of Disease. 5th. The Knife in Uterine Surgery—its uses and abuses. 6th. The Medical Treatment of Old Age. 7th. Rheumatism and Gout.
5. The suggestion and discussion of other topics.

Adjournment at 2:45 p. m.

### EVENING SESSION—7 O'CLOCK.

6. Unfinished and miscellaneous business.

Adjournment at 8:45 precisely.

RECEPTIONS.

Mr. Joel White and Mrs. Dr. Semple, corner of Hull and Jefferson streets, from 9 to 12 p. m.

Mr. and Mrs. John C. Hurter, corner of Madison Avenue and Lawrence street, from 9 to 12 p. m.

Judge and Mrs. David Clopton, corner of Church and Molton streets, from 9 to 12 p. m.

Joie de Vie Club, Perry street, next to Montgomery Theatre, from 10: 30 p. m. to 1 a. m.

FOURTH DAY—FRIDAY, 13TH.

1. Registration.
2. The Association called to order at 9:45 a. m.
3. Unfinished and miscellaneous business.
4. The report of the Board of Censors, including the report of the State Board of Medical Examiners and Committee of Public Health.
5. The revision of the four rolls, including—1st, the revision of the roll of the County Societies; 2d, the revision of the roll of counselors; 3rd, the revision of the roll of correspondents; 4th, the revision of the roll of officers.
6. The election and installation of officers.
7. Unfinished and miscellaneous business.

Adjournment without day.

The President, Dr. Edward H. Sholl, of Birmingham, presided in an admirable manner.

His annual address was devoted to health laws and other matters of general and professional interest.

Contrary to the general custom, everybody connected with this Society is required to work. As an instance, the State is divided into two sections; there is a vice-president from each, who is required to look after the workings of the county societies, boards, etc. The reports this year showed faithful work.

That the Secretary, Dr. T. A. Means, is the right man in the right place is attested by the long number of years he has occupied the position. At this meeting a salary, larger than is paid by any other society, and somewhat commensurate to the onerous duties of the office, was voted him.

Dr. W. C. Jackson, Treasurer, is all that could be desired.

The annual oration, delivered on Tuesday night, on "Health, and Physical Education as a Means of Promoting it," by Dr. B. J. Baldwin, of Montgomery, was able and scholarly, and could it but be read and studied by the people at large would do an incalculable amount of good.

Of the papers read, some were of local interest only, some very practical, and one on an entirely new subject. All were well presented and very interesting.

THE JOURNAL has the promise of several of them and they can speak for themselves. Of the authors of these papers, it is satisfactory to note that a majority were young men—the leaders of the future.

A large part of the third day is devoted to what is known as the "Omnibus Discussion." At every session some one is appointed as leader, whose duty it is to select several subjects and open the discussion at the next meeting. These topics are announced several months in advance, and as a result all are prepared to take a part. Dr. M. C. Baldridge, of Huntsville, was the leader this year, and the omnibus was well freighted with good material.

To a casual observer it would seem that there was too much complication or "red tape" in the detail work of the Association, but anything like an investigation is convincing that it is all necessary to the continued successful working of this well-organized body.

It is a pleasure to announce that every county in the State is now fully organized, with county societies, boards of examiners, health officers, etc., a large majority doing earnest, faithful work.

It is generally conceded that this state of perfection is due in the largest measure to the Grand Senior Censor and State Health Officer, Dr. Jerome Cochrane, an able man, a brilliant worker. It is a pity there is but one of him, else other States might copy the well-administered laws of Alabama.

Dr. M. C. Baldridge was elected President and Dr. B. F. Cross junior Vice-President. The others hold over.

The meeting was wholly successful; the social part elaborate and bountiful.

THE JOURNAL's thanks are due for courtesies extended its representative. They were so numerous it would be invidious to name any one especially. A.

*Montgomery, Ala., April 13th, 1888.*

